# Application of symptom network analysis among patients with gastric cancer: A scoping review

**DOI:** 10.1371/journal.pone.0358940

**Published:** 2026-09-21

**Authors:** Qiu Zhu, Zijing Duan, Yi Dai, Junting Chi, Wangao Guan, Lixuan Dong, Qingjiao Lin, Yingying Fu

**Affiliations:** 1 School of Nursing, Yunnan University of Chinese Medicine, Kunming, Yunnan, China; 2 Department of Nursing, The First People’s Hospital of Yunnan Province, Kunming, Yunnan, China; Hokkaido University: Hokkaido Daigaku, JAPAN

## Abstract

**Objective:**

To map evidence on symptom network analysis in patients with gastric cancer by summarizing study characteristics, commonly used assessment tools, central and bridge symptoms, temporal symptom relationships, and associated factors, and to provide a basis for precision symptom management.

**Methods:**

This scoping review was guided by the Arksey and O’Malley framework and reported in accordance with the PRISMA extension for Scoping Reviews (PRISMA-ScR). PubMed, Web of Science, CINAHL, Cochrane Library, Embase, CNKI, Wanfang Data, VIP, and CBM were systematically searched from database inception to April 1, 2026. Original studies published in Chinese or English that applied symptom network analysis to gastric cancer populations, including eligible mixed cancer populations with gastric cancer patients, during the perioperative period or chemotherapy were included. Literature screening, data extraction, and narrative synthesis were performed.

**Results:**

A total of 9 studies were included. Six symptom assessment tools were identified, with the Chinese version of the MD Anderson Symptom Inventory–Gastrointestinal Cancer Module (MDASI-GI-C) being the most frequently used tool (reported in 7 studies). Nine core symptoms were identified across the included studies, with fatigue being the most frequently reported core symptom (identified in 5 studies). Ten bridge symptoms were identified; however, considerable variation was observed in the identification of bridge symptoms across studies. Longitudinal analyses suggested that phlegm-dampness constitution was associated with subsequent changes in taste, while changes in taste were associated with subsequent appetite loss.

**Conclusions:**

The symptom profiles of patients with gastric cancer are characterized by complexity, dynamic changes across treatment stages, and population heterogeneity. Symptom management strategies may benefit from individualized and stage-specific approaches targeting core symptoms, bridge symptoms, and key symptom pathways. Future research should focus on standardizing symptom assessment tools and assessment time points, conducting multicenter longitudinal studies, and supporting the development and evaluation of interventions for clinical practice.

**Trial registration:**

OSF Registration DOI: https://doi.org/10.17605/OSF.IO/MJAZ3

## 1 Introduction

Gastric cancer is one of the most common malignant tumors of the digestive system worldwide and remains a major global health burden. It ranks among the leading cancers in terms of both incidence and mortality, with approximately 968,000 new cases and 659,000 deaths reported annually worldwide. China contributes substantially to the global burden, accounting for approximately 37.02% of new cases and 39.44% of deaths [1,2]. Surgical treatment and chemotherapy remain the primary therapeutic approaches for gastric cancer. However, patients frequently experience multiple concurrent physical and psychological symptoms during the perioperative period and chemotherapy, with these symptoms often forming interconnected symptom clusters that complicate symptom management [3,4]. Symptom network analysis has emerged as a novel analytical approach for exploring complex symptom interactions. In a symptom network, symptoms are represented as nodes and their statistical associations as edges. Core symptoms are nodes with relatively high centrality and strong connectivity within the network [5], whereas bridge symptoms connect otherwise distinct symptom communities or domains [6]. By identifying central symptoms, bridge symptoms, and relationships among symptom nodes, this method provides new insights into symptom co-occurrence patterns and individualized symptom management strategies [7]. Although research on symptom networks in gastric cancer has gradually increased, variations remain in study populations, assessment instruments, and reported findings. Therefore, guided by Arksey and O’Malley’s scoping review framework [8], this study aims to summarize the application of symptom assessment tools, structural characteristics, and temporal changes in symptom networks among patients with gastric cancer, thereby providing insights for symptom management and future research in this field.

## 2 Materials and methods

### 2.1 Research questions

This scoping review was guided by the following questions: (1) What symptom assessment tools have been used in symptom network studies among patients with gastric cancer? (2) Which core symptoms and bridge symptoms have been identified in gastric cancer symptom networks? (3) What key symptom associations and temporal relationships have been reported among symptoms? (4) What factors are associated with the identification of core symptoms in gastric cancer symptom networks?

### 2.2 Inclusion and exclusion criteria

The inclusion criteria were developed according to the PCC framework (Participants, Concept, and Context) [9]. Studies were eligible if they met the following criteria: (1) Participants: patients diagnosed with gastric cancer, with no restrictions on age, sex, or ethnicity; (2) Concept: studies focusing on symptom network analysis in patients with gastric cancer, including the identification of core symptoms, bridge symptoms, symptom pathways, or network structures; and (3) Context: studies conducted during the perioperative period, chemotherapy period, or postoperative chemotherapy period. Given the limited evidence base, studies of mixed cancer populations were also eligible when gastric cancer patients were explicitly represented in the sample and the study examined symptom networks in a treatment context relevant to gastric cancer. Because gastric-cancer-specific networks could not be isolated in such studies, their findings were treated as indirect evidence and interpreted separately. Studies were excluded if they met any of the following criteria: (1) publications written in languages other than Chinese or English; (2) duplicate publications or studies for which the full text was unavailable; and (3) non-original research articles, including reviews, guidelines, case reports, conference abstracts, and conference proceedings.

### 2.3 Search strategy

A comprehensive literature search was conducted in PubMed, Web of Science, CINAHL (Cumulative Index to Nursing and Allied Health Literature), the Cochrane Library, Embase, China National Knowledge Infrastructure, Wanfang Data, VIP Database, and CBM (Chinese Biomedical Literature Database). The search covered the period from database inception to April 1, 2026. A combination of controlled vocabulary/subject headings and free-text terms was used. Within each concept, synonymous terms were combined with the Boolean operator OR, and the gastric cancer concept was combined with the symptom network analysis concept using AND. Truncation was used where applicable, and the search syntax was adapted to the indexing rules and retrieval functions of each database. In addition, a snowballing approach was applied by manually screening the reference lists of the included studies and relevant articles to identify additional eligible records. The search terms were developed around two main concepts: gastric cancer and symptom network analysis. Chinese-language search terms corresponded to “gastric tumour”, “malignant gastric tumour”, “gastric cancer”, “symptom network”, “network analysis”, and “core symptoms”. English search terms included “gastric cancer*”, “stomach cancer*”, “gastric neoplasm*”, “stomach neoplasm*”, “malignant gastric neoplasm*”, “malignant stomach neoplasm*”, “cancer of the stomach”, “familial diffuse gastric cancer”, “symptom network*”, “network of symptoms”, “symptom network structure”, “network analysis”, “symptom network analysis”, “core symptom*”, and “key symptom*”. The complete PubMed strategy, including MeSH and title/abstract terms and Boolean combinations, is presented in Table 1.

**Table 1:** PubMed search strategy.

Step	Search strategy
#1	“Stomach Neoplasms”[Mesh]
#2	“Gastric Cancer”[Title/Abstract] OR “Stomach Cancer”[Title/Abstract] OR “Gastric Neoplasm”[Title/Abstract] OR “Stomach Neoplasm”[Title/Abstract] OR “Malignant Gastric Neoplasm”[Title/Abstract] OR “Malignant Stomach Neoplasm”[Title/Abstract] OR “Cancer of Stomach”[Title/Abstract] OR “Cancer of the Stomach”[Title/Abstract] OR “Gastric Cancer, Familial Diffuse”[Title/Abstract]
#3	#1 OR #2
#4	“Symptom Network”[Title/Abstract] OR “Network of Symptoms”[Title/Abstract] OR “Symptom Network Structure”[Title/Abstract] OR “Network Analysis”[Title/Abstract] OR “Symptom Network Analysis”[Title/Abstract] OR “Core Symptom”[Title/Abstract] OR “Key Symptom”[Title/Abstract]
#5	#3 AND #4

### 2.4 Literature screening and data extraction

Duplicate records were removed using Zotero 7.0. Two researchers, the first and second authors, independently screened all retrieved studies based on the predefined inclusion and exclusion criteria. In the first stage, titles and abstracts were reviewed to exclude clearly irrelevant studies. In the second stage, the full texts of potentially eligible studies were assessed. Disagreements were discussed with a third researcher, the corresponding author, until consensus was reached. The final included studies were then confirmed. Data were extracted using a standardized Microsoft Excel form. The extracted items included first author, publication year, country, study design, sample size, study population, assessment time points, assessment tools, and main findings. Methodological characteristics of the network analyses were also charted, including network model, software/packages, centrality indicators, bridge indicators, temporal indicators, and reporting of network stability and estimation accuracy.

### 2.5 Reporting guideline and methodological quality appraisal

This review was reported in accordance with the Preferred Reporting Items for Systematic Reviews and Meta-Analyses extension for Scoping Reviews (PRISMA-ScR), and the completed checklist is provided in S1 File. A formal risk-of-bias or methodological quality appraisal was intentionally not undertaken because the aim of this scoping review was to map the breadth, characteristics, methods, and findings of the available evidence rather than to estimate pooled intervention effects or grade certainty of evidence. Instead, methodological features directly relevant to the interpretation of symptom network studies, including network estimation approaches, centrality and bridge indicators, temporal indicators, and stability/accuracy reporting, were systematically charted.

### 2.6 Ethical review

As this review was conducted solely on the basis of published studies, it did not involve human participants, individual-level data, or animal experiments. Ethical approval was therefore not required. The review protocol has been registered with the Open Science Framework under DOI: https://doi.org/10.17605/OSF.IO/MJAZ3

## 3 Results

### 3.1 Results of literature screening

A total of 669 records were identified through the initial search. Following eligibility screening based on the predefined inclusion and exclusion criteria, 9 studies [10–18] were ultimately included in this review. The literature screening process is shown in S1 Fig.

### 3.2 Basic characteristics of the included studies

Among the 9 included studies, 5 were cross-sectional studies [11–14,16] and 4 were longitudinal studies [10,15,17,18]. Seven studies were conducted in China [10–16], and two were conducted in South Korea [17,18]. The study populations mainly comprised patients receiving chemotherapy for gastric cancer, patients undergoing postoperative chemotherapy, and patients in the perioperative period of gastric cancer treatment. In addition, two studies [16,18] included mixed cancer populations with gastric cancer patients. These studies were retained to map potentially relevant evidence but were treated as indirect evidence because gastric-cancer-specific symptom networks were not reported. The detailed characteristics of the included studies are presented in Table 2.

**Table 2:** Basic characteristics of the included studies (n = 9).

First Author	Year	Country	Design	Sample Size	Assessment Time	Participants	Assessment Tools
Li Siyu [10]	2025	China	Longitudinal	386	After the 2nd, 4th, and 6th chemotherapy cycles	Patients receiving chemotherapy for gastric cancer	MDASI-GI-C; TCM Constitution Scale
Zou Yanling [11]	2024	China	Cross-sectional	340	Not reported	Patients receiving chemotherapy for gastric cancer	MDASI-GI-C; TCM Constitution Scale
Ge Jiaoyu [12]	2025	China	Cross-sectional	269	Postoperative day 7	Postoperative gastric cancer patients	MDASI-GI-C
Zou [13]	2024	China	Cross-sectional	355	Second day of chemotherapy	Postoperative chemotherapy patients with gastric cancer	MDASI-GI-C; TCM Constitution Scale
Li [14]	2024	China	Cross-sectional	677	From chemotherapy initiation to discharge	Postoperative chemotherapy patients with gastric cancer	MDASI-GI-C
Li [15]	2025	China	Longitudinal	379	After the 1st, 3rd, and 6th chemotherapy cycles	Postoperative chemotherapy patients with gastric cancer	MDASI-GI-C
Wang [16]	2025	China	Cross-sectional	202 (GC = 62)	Not reported	Patients with gastrointestinal cancer	MDASI-GI-C
Shim [17]	2021	South Korea	Longitudinal	256	1 week before surgery, 1 week after surgery, 3–6 months after surgery	Perioperative gastric cancer patients	MDASI; HADS; FACT-Ga
Rha [18]	2021	South Korea	Longitudinal	249 (GC = 83)	Before and after chemotherapy	Patients with mixed cancer types receiving chemotherapy	20-item Symptom Severity Scale

Note: MDASI-GI-C, Chinese version of the M. D. Anderson Symptom Inventory Gastrointestinal Cancer Module; MDASI, M. D. Anderson Symptom Inventory; HADS, Hospital Anxiety and Depression Scale; FACT-Ga, Functional Assessment of Cancer Therapy–Gastric Cancer Module; TCM Constitution Scale, Classification and Assessment of Traditional Chinese Medicine Constitution; GC, gastric cancer.

#### 3.2.1 Symptom assessment tools.

A total of six relevant assessment instruments were used across the included studies. The Chinese version of the MD Anderson Symptom Inventory–Gastrointestinal Cancer Module (MDASI-GI-C) was the most frequently used instrument (n = 7) [10–16]. The remaining instruments included the Classification and Determination of Traditional Chinese Medicine Constitution (n = 3) [10,11,13], the MD Anderson Symptom Inventory (MDASI) [17], the Hospital Anxiety and Depression Scale (HADS) [17], the Functional Assessment of Cancer Therapy–Gastric (FACT-Ga) [17], and the 20-item Symptom Severity Scale [18].

#### 3.2.2 Timing of symptom assessment.

Perioperative studies assessed symptoms at multiple time points before and after surgery (n = 2) [12,17], including 1 week before surgery, 1 week after surgery, 7 days postoperatively, and 3–6 months after surgery. Chemotherapy-related studies primarily evaluated symptoms before and after chemotherapy or across different chemotherapy cycles (n = 5) [10,13–15,18], including assessments after the first, second, third, fourth, and sixth chemotherapy cycles, on the second day of chemotherapy, or from chemotherapy initiation to hospital discharge. In addition, two cross-sectional studies did not report detailed symptom assessment time points [11,16].

### 3.3 Main findings

A total of 7 studies [11–14,16–18] explicitly identified core symptoms across different treatment stages, while 3 studies [11,15,17] reported bridge symptoms. Three studies [11–13] explored factors associated with core symptoms, and 2 studies [10,15] examined temporal relationships among symptoms. The detailed characteristics are presented in Table 3. All 9 studies [10–18] used R software for network estimation and visualization. Among them, 5 studies [11,12,14,17,18] used strength centrality to identify core symptoms, and 3 studies [12,15,16] reported the stability and accuracy of their network results. The detailed methodological characteristics are presented in Table 4.

**Table 3:** Main findings of the included studies.

First Author	Core Symptoms/Clusters	Bridge Symptoms	Temporal Relationships	Other Findings
Li Siyu [10]	—	—	T1 → T2: Blood-stasis→fatigue; Yang-deficiency→sadness; sadness→fatigue (strongest); highest out/in: sadness/Qi-stagnation.T2 → T3: Phlegm-dampness→taste changes; taste changes→loss of appetite (strongest); highest out/in: taste changes/loss of appetite.	Phlegm-dampness constitution predicted taste changes; sadness predicted fatigue
Zou Yanling [11]	Sadness, taste changes, vomiting	Sleep disturbance, loss of appetite, somnolence, taste changes	—	Taste changes–loss of appetite and nausea–vomiting showed strong associations
Ge Jiaoyu [12]	Vomiting, fatigue, distress	—	—	Age, sex, surgical approach, hemoglobin, prealbumin, education level, and complications were associated with symptoms
Zou [13]	Taste changes, vomiting, loss of appetite	—	—	Taste changes–loss of appetite and nausea–vomiting showed strong associations
Li [14]	Distress in the low-symptom-burden group; loss of appetite in the moderate-symptom-burden group; fatigue in the high-symptom-burden group; sadness in the overall sample.	—	—	Taste changes–loss of appetite, sadness–distress, and nausea–vomiting were closely connected
Li [15]	—	Dry mouth in early chemotherapy; abdominal distension in later stages	T1 → T2: Dry mouth→others; others→distress; vomiting→loss of appetite strongest.T2 → T3: Abdominal distension→others; others→loss of appetite; strongest: abdominal distension→nausea and taste changes→loss of appetite.	Dynamic symptom changes across chemotherapy cycles
Wang [16]	Distress; psychological symptom cluster	—	—	Psychological symptoms were central in the network
Shim [17]	Preoperative: shortness of breath, fatigue; 1 week postoperative: fatigue; 3–6 months postoperative: physical health, gastric cancer–related concerns. Distress was central at all time points.	Anxiety, depression, emotional well-being, physical health	—	Psychological symptoms connected symptoms with quality of life
Rha [18]	Fatigue	—	—	Anxiety–depression, fatigue–somnolence, and loss of appetite–taste changes were closely linked

**Table 4:** Network analysis methods and reporting characteristics.

First Author	Network Method	Software/Packages	Core Indicators	Bridge Indicators	Temporal Indicators	Stability Reporting
Li Siyu [10]	Cross-lagged panel network	R; glmnet; qgraph	—	—	Cross-lagged coefficients; in/out strength	CS and CI were not reported
Zou Yanling [11]	Cross-sectional network	R; igraph	Strength centrality	Bridge strength centrality	—	CS = 0.67; CI not reported
Ge Jiaoyu [12]	Cross-sectional network	R; qgraph	Strength centrality	—	—	CS = 0.589; bootstrap CI reported
Zou [13]	Cross-sectional network	R; qgraph; ggplot2	Strength and closeness centrality	—	—	CS = 0.825; CI not reported
Li [14]	Stratified network comparison	R; qgraph	Strength centrality	—	—	Not reported
Li [15]	Cross-lagged panel network	R; glmnet; qgraph	—	Bridge strength centrality	Cross-lagged coefficients	CS = 0.517; bootstrap CI reported
Wang [16]	Cross-sectional network	R; qgraph; Gephi	Strength and closeness centrality	—	—	CS = 0.441; bootstrap CI reported
Shim [17]	Multi-time-point network	R; qgraph; networktools	Strength centrality	Bridge expected influence	—	CS reported; CI not reported
Rha [18]	Repeated contemporaneous networks	R; qgraph; Walktrap	Strength centrality	—	—	CS reported; CI not reported

Note: CS, correlation stability coefficient; CI, bootstrap 95% confidence interval; “Not reported” indicates that the relevant information was not provided in the original article.

#### 3.3.1 Core symptoms.

A total of 7 studies identified core symptoms among patients with gastric cancer or mixed cancer populations including patients with gastric cancer [11–14,16–18]. Regarding the frequency of identification, fatigue was reported as a core symptom in 5 studies, followed by pain and vomiting (3 studies each), and sadness, altered taste, and loss of appetite (2 studies each). Shortness of breath and certain quality-of-life-related symptoms were each reported in 1 study. Among these symptoms, fatigue was the most frequently identified core symptom and was repeatedly observed across different treatment phases, including among postoperative gastric cancer patients, patients receiving postoperative chemotherapy with high symptom burden, perioperative gastric cancer patients, and patients undergoing chemotherapy in mixed cancer populations [12,14,17,18]. The core symptoms varied across treatment phases. Among gastric cancer patients on postoperative day 7, vomiting, fatigue, and pain were identified as the primary core symptoms [12], whereas altered taste, vomiting, and loss of appetite were identified as core symptoms among patients receiving postoperative chemotherapy [13]. Furthermore, differences in core symptoms were observed among patient subgroups with different symptom burden levels [14].

#### 3.3.2 Bridge symptoms.

Three studies identified bridge symptoms in symptom networks among patients with gastric cancer [11,15,17]. The identified bridge symptoms included sleep disturbances, loss of appetite, somnolence, altered taste, dry mouth, abdominal distension, anxiety, depression, emotional well-being, and physical health. Zou Yanling et al. [11] reported that sleep disturbances, loss of appetite, somnolence, and altered taste were identified as bridge symptoms among patients with gastric cancer undergoing chemotherapy. Li et al. [15] found that dry mouth served as a bridge symptom during the early stage of postoperative chemotherapy, whereas abdominal distension emerged as a bridge symptom during the middle and late stages of chemotherapy. Shim et al. [17] investigated symptom–quality of life associations and found that anxiety, depression, and emotional well-being played bridge roles before surgery, at 1 week postoperatively, and at 3–6 months postoperatively. Considerable variation was observed in the bridge symptoms identified across these three studies.

#### 3.3.3 Temporal relationships among symptoms.

Two studies further explored temporal relationships among symptoms in patients with gastric cancer [10,15]. Using cross-lagged network analysis, Li Siyu et al. [10] found that the path from sadness to fatigue showed a strong temporal association between the second and fourth chemotherapy cycles, whereas the path from altered taste to loss of appetite showed a strong association between the fourth and sixth cycles. Li et al. [15] reported that “vomiting → loss of appetite” showed the strongest positive cross-lagged association from the first to the third postoperative chemotherapy sessions, while “abdominal distension → nausea” showed the strongest cross-lagged association from the third to the sixth sessions; meanwhile, “altered taste → loss of appetite” showed the strongest cross-lagged association. Together, these studies suggest that the relationship between altered taste and loss of appetite may represent a relatively consistent temporal association across chemotherapy phases. Because both studies were observational, these cross-lagged paths should be interpreted as time-ordered statistical associations rather than evidence of causal effects.

#### 3.3.4 Strongly connected symptom pairs.

Several studies reported symptom pairs with strong associations within gastric cancer symptom networks, primarily involving gastrointestinal symptoms, eating-related symptoms, and psychological and emotional symptoms [11,13–18]. Among these, “altered taste–loss of appetite” and “nausea–vomiting” were repeatedly identified as strongly connected symptom pairs across multiple studies, reflecting important symptom interactions related to gastric cancer and its treatment [11,13–15,18]. Strong associations were also observed among psychological and emotional symptoms; for example, “grief–distress” and “anxiety–depression” repeatedly emerged across different studies [11,14,17,18]. In a study involving patients with gastrointestinal cancer, Wang et al. [16] reported that “distress–grief,” “grief–numbness,” and “pain–loss of appetite” showed the strongest associations, and that the psychological and emotional symptom cluster represented a central component of the symptom network.

#### 3.3.5 Association between Traditional Chinese Medicine constitution and symptoms.

Three included studies incorporated Traditional Chinese Medicine (TCM) constitution factors into symptom network analyses among patients with gastric cancer undergoing chemotherapy [10,11,13]. TCM constitution types were associated with chemotherapy-related symptom patterns among patients with gastric cancer. In particular, Qi-deficiency, Phlegm-dampness, Blood-stasis, Yang-deficiency, and Qi-stagnation constitutions showed cross-sectional or time-ordered statistical associations with specific symptom nodes or symptom relationships within the network. Based on cross-lagged network analysis, Li Siyu et al. [10] found that Blood-stasis constitution showed a positive temporal association with subsequent fatigue, Yang-deficiency constitution was associated with subsequent sadness, and Phlegm-dampness constitution was associated with subsequent altered taste. In addition, the Harmonious constitution showed a tendency to transition toward the Qi-stagnation constitution. Zou Yanling et al. [11] reported that Qi-deficiency and Phlegm-dampness constitutions were positively associated with sadness severity, while Phlegm-dampness constitution was positively associated with altered taste severity. Overall, current evidence suggests a relatively consistent association between Phlegm-dampness constitution and altered taste among patients with gastric cancer undergoing chemotherapy. These findings are associative and hypothesis-generating; they do not demonstrate that TCM constitution causes subsequent symptom changes.

#### 3.3.6 Network analysis methods and reporting characteristics.

Five studies employed cross-sectional or stratified network analyses [11–14,16], two used cross-lagged panel network analysis [10,15], and two constructed longitudinal symptom networks to examine temporal changes during surgery or chemotherapy [17,18]. All studies used R software for network estimation and visualization, with qgraph being the most commonly used package. Strength centrality was the primary indicator for identifying core symptoms, and most studies reported network stability or bootstrap analyses. However, variations in centrality metrics and stability reporting methods limited the comparability of findings across studies. Detailed characteristics of the network analysis methods and reporting features are presented in Table 4.

## 4 Discussion

### 4.1 Heterogeneity in the identification of core symptoms among patients with gastric cancer

Core symptoms are symptom nodes with relatively high centrality and strong connectivity to other nodes in the network; identifying these symptoms may help prioritize candidates for targeted symptom management [5]. However, core symptoms identified among patients with gastric cancer varied across studies, likely due to differences in treatment stages, assessment instruments, time points, and symptom nodes analyzed. Although some overlap existed among studies, no universally applicable core symptoms have been established. For example, among patients receiving postoperative chemotherapy, Zou et al. [13] identified altered taste as a core symptom, whereas Li et al. [14] identified sadness as the most prominent symptom. Differences in assessment tools may also contribute to these variations, as the MDASI-GI-C emphasizes gastrointestinal symptoms, while the MDASI incorporates psychological symptoms and quality-of-life domains. In addition, core symptoms changed across treatment phases; vomiting, fatigue, and pain were prominent after surgery [12], whereas altered taste, vomiting, and loss of appetite were more common during chemotherapy [13]. Overall, fatigue, vomiting, altered taste, loss of appetite, and pain warrant attention in gastric cancer symptom networks; however, no core symptom has been consistently identified across all treatment phases. Future studies should standardize assessment tools, time points, inclusion criteria, and network metrics, and conduct stage-specific and stratified analyses to improve the stability and comparability of core symptom identification.

### 4.2 Limited evidence on bridge symptoms and factors associated with core symptoms

Bridge symptoms connect different symptom clusters or dimensions and may help identify potential targets for limiting cross-domain symptom connectivity within the network [6]. However, among the nine included studies, only three reported bridge symptoms, and the identified bridge symptoms varied due to differences in treatment stages, assessment tools, and inclusion criteria. Zou Yanling et al. [11] found that sleep disturbances, loss of appetite, and somnolence acted as bridge symptoms in patients undergoing chemotherapy for gastric cancer, whereas Li et al. [15] reported that dry mouth was a bridge symptom during early postoperative chemotherapy and transitioned to abdominal distension during middle and late stages. These findings suggest that bridge symptoms may show stage-specific changes, although current evidence remains limited. Future studies should standardize symptom assessment tools, inclusion criteria, and assessment time points, and conduct longitudinal network analyses across treatment phases to identify relatively stable bridge symptoms and guide phased symptom interventions. Research on factors associated with core symptoms also remains limited, with current evidence mainly focusing on vomiting, fatigue, distress, and altered taste. Ge Jiaoyu et al. [12] found that age, sex, and surgical approach were associated with postoperative vomiting; hemoglobin and prealbumin levels with postoperative fatigue; and education level and postoperative complications with distress. Studies by Zou Yanling et al. [11], Zou et al. [13], and Li Siyu et al. [10] suggested an association between Phlegm-dampness constitution and altered taste, with Li Siyu et al. [10] further identifying a temporal association between Phlegm-dampness constitution and subsequent taste changes. According to Traditional Chinese Medicine theory, Phlegm-dampness constitution is associated with impaired spleen-stomach transformation and transportation, abnormal fluid metabolism, and internal accumulation of phlegm and dampness. Clinically, this may manifest as a sticky sensation in the mouth, thick and greasy tongue coating, and poor appetite [19]. A thick and greasy tongue coating has been proposed as a possible correlate of altered taste perception, while chemotherapy-related injury to taste and olfactory sensory cells provides a separate biomedical explanation for taste changes [20]. These mechanisms should be regarded as theoretical explanations rather than causal interpretations of the observed network associations. Accordingly, taste changes may warrant closer monitoring in patients with gastric cancer undergoing chemotherapy, including those classified as having Phlegm-dampness constitution. Within culturally appropriate TCM-informed care, strategies such as dietary guidance, oral care, moderate exercise, acupoint massage, and auricular seed therapy [21] may be considered as supportive approaches; however, the network studies included in this review do not establish that modifying TCM constitution improves taste perception or eating status. Prospective intervention studies are needed to test these hypotheses.

TCM constitution also raises a distinct issue of cross-cultural transferability. It is a culturally embedded diagnostic and classification construct without direct conceptual or measurement equivalents in many non-Chinese healthcare systems. Therefore, associations involving TCM constitution should not be assumed to generalize to other cultural or clinical settings simply because the symptom nodes themselves are common across populations. Testing transferability would require explicit cross-cultural operationalization and validation of the constitution construct, followed by replication of the observed symptom-network associations in more diverse populations.

### 4.3 Limited evidence on longitudinal symptom relationships and potential value of strongly connected symptom pairs for intervention

Temporal relationships among symptoms provide information on time-ordered statistical associations between earlier and later symptoms, helping to characterize symptom evolution patterns and generate hypotheses for proactive symptom management [22]. Among the four longitudinal studies included in this review, only two applied cross-lagged network analysis to examine temporal symptom relationships, indicating that current evidence remains limited. Although Li Siyu et al. [10] and Li et al. [15] both explored cross-temporal symptom associations, differences in assessment time points and included symptoms make it difficult to establish consistent conclusions. Future studies should standardize longitudinal assessment schedules, conduct continuous follow-up across treatment phases, and replicate temporal symptom associations through multicenter, large-sample studies. Despite limited evidence regarding temporal relationships, several strongly connected symptom pairs have been repeatedly identified and may represent potential intervention targets. Altered taste and loss of appetite showed consistent associations across multiple studies [11,13–15,18], and cross-lagged network analyses further showed that altered taste was temporally associated with subsequent loss of appetite. Therefore, early assessment of taste changes during chemotherapy, combined with oral care, dietary adjustment, and nutritional support, could be evaluated prospectively as a strategy for reducing later appetite loss and malnutrition risk. Nausea–vomiting was also repeatedly identified as a stable gastrointestinal symptom association [11,13,14], highlighting the importance of early prevention and continuous management of chemotherapy-induced nausea and vomiting. In addition, psychological symptom associations, particularly grief–distress, were repeatedly observed [11,14,16], suggesting that symptom management should incorporate psychological screening and emotional and social support alongside physical symptom control. Overall, symptom management for gastric cancer should move beyond individual symptom control toward interventions targeting key symptom interactions, with comprehensive and continuous management focusing on gastrointestinal symptoms, nutritional status, and psychological well-being.

Network analysis provides intervention-relevant information that differs structurally from conventional symptom cluster analysis. Conventional symptom cluster methods primarily identify groups of symptoms that co-occur and therefore support coordinated management of symptom sets [23], whereas network analysis additionally distinguishes high-connectivity central symptoms, bridge symptoms linking different symptom communities, and, in longitudinal models, time-ordered associations between symptoms. These features can generate more specific hypotheses about intervention prioritization and sequencing: central symptoms may be considered candidate high-connectivity targets, whereas bridge symptoms may be considered when the goal is to limit propagation of symptom burden across domains. For example, Li et al. [15] found that altered taste was temporally associated with subsequent loss of appetite. A conventional cluster analysis would support co-management of these eating-related symptoms, whereas the cross-lagged network suggests that early management of taste change could be tested as an upstream strategy before appetite deterioration occurs. The same study identified dry mouth as a bridge symptom in early chemotherapy and abdominal distension as a bridge symptom in later cycles, suggesting that candidate intervention targets may change across treatment phases. These implications remain hypothesis-generating because centrality, bridge status, and cross-lagged associations do not by themselves establish causal intervention effects.

### 4.4 Similarities and differences between mixed cancer studies and gastric cancer-specific studies

Mixed cancer studies and gastric cancer-specific studies have demonstrated both shared symptom patterns and disease-specific differences. Wang et al. [16] identified distress as a core symptom in a mixed gastrointestinal cancer population, with psychological and emotional symptoms forming a central component of the network. This finding was consistent with the importance of grief, distress, anxiety, and depression reported in gastric cancer-specific studies [11,14,17]. Rha et al. [18] identified fatigue as the most central symptom among patients with various cancer types undergoing chemotherapy and reported strong associations between anxiety–depression, fatigue–somnolence, and loss of appetite–altered taste. These findings partially correspond with the central role of fatigue and the altered taste–loss of appetite association observed in gastric cancer studies [11–15,17]. These similarities may reflect shared symptom patterns related to cancer and chemotherapy, but they do not establish that the underlying symptom-network structure is identical across cancer types. However, gastric cancer-specific studies placed greater emphasis on gastrointestinal and disease-specific symptoms, including vomiting, altered taste, loss of appetite, abdominal distension, and gastric cancer-related concerns. In the two mixed cancer studies, gastric cancer patients accounted for only 62/202 and 83/249 participants, respectively, and no separate symptom networks were constructed for gastric cancer subgroups. Therefore, findings from mixed cancer studies should be interpreted as indirect evidence with limited applicability to gastric cancer populations.

### 4.5 Network analysis modeling and heterogeneity in reporting

In addition to differences in treatment phases, assessment tools, and study populations, variations in network modeling approaches represent an important source of heterogeneity. Cross-sectional networks estimate conditional associations among symptoms at a single time point but cannot determine temporal sequences. In contrast, cross-lagged panel networks can estimate time-ordered associations but may be influenced by assessment intervals, regularization parameters, and node selection; such associations do not establish causality. Multi-time-point network analyses used by Shim et al. [17] and Rha et al. [18] captured changes in network structures across treatment stages but could not fully account for individual-level temporal effects. Methodological differences may also affect the identification of core and bridge symptoms. While Wang et al. [16] used Gephi for clustering and network analysis, most studies applied R-based regularized network models; differences in algorithms, centrality metrics, and standardization procedures may contribute to inconsistent symptom rankings. Therefore, repeated identification of symptoms such as fatigue and distress should not be interpreted solely as evidence of cross-study consistency. The interpretation of centrality results also depends on network stability and estimation accuracy. Although some studies reported strength centrality stability coefficients (CS-coefficients) above 0.50, indicating relatively stable rankings, lower stability estimates for closeness or betweenness centrality at certain time points suggest that these results should be interpreted cautiously. In addition, incomplete reporting of bootstrap confidence intervals for edge weights limited evaluation of estimation precision. Future studies should standardize reporting of network models, regularization parameters, edge-weight confidence intervals, centrality stability coefficients, and difference tests, and prioritize interpretation of indicators supported by adequate stability to improve transparency, reproducibility, and comparability across studies.

## 5 Conclusions

Symptom network findings in patients with gastric cancer vary across treatment stages and patient populations and differ in relation to assessment timing, symptom assessment tools, and patient characteristics. Fatigue, vomiting, altered taste, loss of appetite, and distress were repeatedly identified across several studies, suggesting that these symptoms may represent important targets for symptom management. Relatively consistent associations were also observed between altered taste and loss of appetite, nausea and vomiting, and sadness and distress. These findings support consideration of more comprehensive symptom management that addresses key symptoms and their interconnections, while the effectiveness of network-informed intervention targets requires prospective evaluation.

However, current evidence regarding bridge symptoms, factors associated with core symptoms, and temporal relationships among symptoms remains limited, and consistent conclusions have not yet been established. The present review also has several limitations. Only nine studies were included, seven were conducted in China, and the remaining two were conducted in South Korea, limiting the geographic and cross-cultural generalizability of the evidence. Two studies involved mixed cancer populations and did not report gastric-cancer-specific networks; their findings were therefore indirect and should not be assumed to represent gastric cancer alone. In addition, TCM constitution is a culturally embedded construct without direct equivalents in many non-Chinese healthcare and assessment systems, creating a distinct limitation in the conceptual and measurement transferability of findings involving constitution variables. Considerable methodological heterogeneity was observed across studies in study design, symptom assessment tools, assessment time points, node selection, network estimation methods, centrality measures, and reporting of stability and accuracy, which limited direct comparison across studies. A formal risk-of-bias or methodological quality appraisal was intentionally not conducted because the review aimed to map the available evidence; consequently, the methodological quality of individual studies was not used to weight the synthesis or conclusions.

Future research should standardize symptom assessment instruments, assessment schedules, and the reporting of network indicators. Multicenter longitudinal studies with larger and more culturally diverse samples are needed across the perioperative, chemotherapy, and rehabilitation phases. Stratified analyses according to symptom burden and patient characteristics should also be strengthened to improve the reliability and clinical relevance of identified key symptoms and symptom pathways. In clinical practice, gastrointestinal symptoms, nutritional intake, fatigue, and psychological and emotional changes should be continuously assessed. Stage-specific and holistic management strategies should be prospectively evaluated to determine whether network-informed prioritization improves outcomes beyond conventional single-symptom or symptom-cluster management.

## Supporting information

S1 Fig Flowchart of literature screening. (DOCX)

S1 File PRISMA-ScR Checklist. (PDF)

S2 File Search Strategies 8 Databases. (DOCX)
