# Supplementary material for: Application of symptom network analysis among patients with gastric cancer: A scoping review
Source: PLoS One. 2026 Sep 21;21(9):e0358940. doi: 10.1371/journal.pone.0358940 (PMC13592616; doi:10.1371/journal.pone.0358940)
Supplement: Supplementary file 3 [file pone.0358940.s003.docx]

**Complete Search Strategies for Eight Databases Other Than PubMed**

**1. Web of Science**

**Search string**

TS=("gastric cancer*" OR "stomach cancer*" OR "gastric neoplasm*" OR "stomach neoplasm*" OR "malignant gastric neoplasm*" OR "malignant stomach neoplasm*" OR "cancer of the stomach" OR "familial diffuse gastric cancer")

TS=("symptom network*" OR "network of symptoms" OR "symptom network structure" OR "network analysis" OR "symptom network analysis" OR "core symptom*" OR "key symptom*")

#1 AND #2

**2. CINAHL**

**Search string**

MH "Stomach Neoplasms+"

TI ("gastric cancer*" OR "stomach cancer*" OR "gastric neoplasm*" OR "stomach neoplasm*" OR "malignant gastric neoplasm*" OR "malignant stomach neoplasm*" OR "cancer of the stomach" OR "familial diffuse gastric cancer") OR AB ("gastric cancer*" OR "stomach cancer*" OR "gastric neoplasm*" OR "stomach neoplasm*" OR "malignant gastric neoplasm*" OR "malignant stomach neoplasm*" OR "cancer of the stomach" OR "familial diffuse gastric cancer") OR KW ("gastric cancer*" OR "stomach cancer*" OR "gastric neoplasm*" OR "stomach neoplasm*" OR "malignant gastric neoplasm*" OR "malignant stomach neoplasm*" OR "cancer of the stomach" OR "familial diffuse gastric cancer")

S1 OR S2

TI ("symptom network*" OR "network of symptoms" OR "symptom network structure" OR "network analysis" OR "symptom network analysis" OR "core symptom*" OR "key symptom*") OR AB ("symptom network*" OR "network of symptoms" OR "symptom network structure" OR "network analysis" OR "symptom network analysis" OR "core symptom*" OR "key symptom*") OR KW ("symptom network*" OR "network of symptoms" OR "symptom network structure" OR "network analysis" OR "symptom network analysis" OR "core symptom*" OR "key symptom*")

S3 AND S4

**3. Cochrane Library**

**Search string**

MeSH descriptor: [Stomach Neoplasms] explode all trees

("gastric cancer*" OR "stomach cancer*" OR "gastric neoplasm*" OR "stomach neoplasm*" OR "malignant gastric neoplasm*" OR "malignant stomach neoplasm*" OR "cancer of the stomach" OR "familial diffuse gastric cancer"):ti,ab,kw

#1 OR #2

("symptom network*" OR "network of symptoms" OR "symptom network structure" OR "network analysis" OR "symptom network analysis" OR "core symptom*" OR "key symptom*"):ti,ab,kw

#3 AND #4

**4. Embase**

**Search string**

'stomach tumor'/exp

('gastric cancer*' OR 'stomach cancer*' OR 'gastric neoplasm*' OR 'stomach neoplasm*' OR 'malignant gastric neoplasm*' OR 'malignant stomach neoplasm*' OR 'cancer of the stomach' OR 'familial diffuse gastric cancer'):ti,ab,kw

#1 OR #2

('symptom network*' OR 'network of symptoms' OR 'symptom network structure' OR 'network analysis' OR 'symptom network analysis' OR 'core symptom*' OR 'key symptom*'):ti,ab,kw

#3 AND #4

**5. China National Knowledge Infrastructure (CNKI)**

**Search string**

(SU='gastric tumor' OR SU='malignant gastric tumor' OR SU='gastric cancer')

(SU='symptom network' OR SU='network analysis' OR SU='core symptom')

#1 AND #2

(SU='gastric tumor' OR SU='malignant gastric tumor' OR SU='gastric cancer') AND (SU='symptom network' OR SU='network analysis' OR SU='core symptom')

**6. Wanfang Data**

**Search string**

Subject: ('gastric tumor' OR 'malignant gastric tumor' OR 'gastric cancer')

Subject: ('symptom network' OR 'network analysis' OR 'core symptom')

#1 AND #2

Subject: ('gastric tumor' OR 'malignant gastric tumor' OR 'gastric cancer') AND Subject: ('symptom network' OR 'network analysis' OR 'core symptom')

**7. VIP Database**

**Search string**

(SU='gastric tumor' OR SU='malignant gastric tumor' OR SU='gastric cancer')

(SU='symptom network' OR SU='network analysis' OR SU='core symptom')

#1 AND #2

(SU='gastric tumor' OR SU='malignant gastric tumor' OR SU='gastric cancer') AND (SU='symptom network' OR SU='network analysis' OR SU='core symptom')

**8. Chinese Biomedical Literature Database (CBM/SinoMed)**

**Search string**

Common fields (intelligent search): 'gastric tumor' OR 'malignant gastric tumor' OR 'gastric cancer'

Common fields (intelligent search): 'symptom network' OR 'network analysis' OR 'core symptom'

#1 AND #2
