# Supplementary material for: Application of symptom network analysis among patients with gastric cancer: A scoping review
Source: PLoS One. 2026 Sep 21;21(9):e0358940. doi: 10.1371/journal.pone.0358940 (PMC13592616; doi:10.1371/journal.pone.0358940)
Supplement: Supplementary file 4 [file pone.0358940.s005.docx]

**Response to Reviewers**

**Manuscript title:**

Application of symptom network analysis in symptom management among patients with gastric cancer: A scoping review

Manuscript ID:
PONE-D-26-22137

Dear Editor and Reviewers,

We sincerely thank you for the time and effort you devoted to carefully reviewing our manuscript and for providing professional, detailed, and constructive comments and suggestions. The issues raised are highly valuable and have not only helped us identify the limitations and deficiencies in the original manuscript but have also enabled us to further improve the research content, refine the structure of the manuscript, provide additional analyses and explanations, and enhance its scientific rigor, clarity, and readability. We have carefully considered and thoroughly discussed all the comments provided by the Editor and Reviewers and have revised the manuscript accordingly. In the following sections, we provide a detailed, point-by-point response to each comment and describe the corresponding revisions, together with their specific locations in the revised manuscript.

==================================================
**Additional Improvements to the Manuscript**
==================================================

Based on the comments and suggestions provided by the Editor and Reviewers, in addition to addressing each comment individually, we also made several further improvements to the manuscript.

First, to make the title more concise and accurate and to ensure that it better reflects the actual scope of the review, we changed the original title from “Application of symptom network analysis in symptom management among patients with gastric cancer: A scoping review” to “Application of symptom network analysis among patients with gastric cancer: A scoping review.”

Second, several studies included in this review investigated the relationships between symptoms and traditional Chinese medicine constitution among patients with gastric cancer. In addition, one reviewer raised an important question regarding whether findings related to traditional Chinese medicine constitution are applicable to populations in other countries and regions. Therefore, we added further information on this topic to both the Results and Discussion sections and provided a more cautious and comprehensive explanation of its scope of applicability, cultural context, and the generalizability of the relevant findings.

Furthermore, we carefully reviewed the entire manuscript again and revised statements that were inaccurate, unclear, or inappropriate to further improve the scientific rigor, coherence, and readability of the manuscript.

Location of revision:

Manuscript title: Title page, Page 1, Lines 1–2. The title of the manuscript was revised to “Application of symptom network analysis among patients with gastric cancer: A scoping review.”

Content related to Traditional Chinese Medicine constitution: Results section, subsection “3.3.5 Association between Traditional Chinese Medicine constitution and symptoms,” Pages 10–11, Lines 209–224; Discussion section, subsection “4.2 Limited evidence on bridge symptoms and factors associated with core symptoms,” Pages 13–14, Lines 272–287.

Language and wording throughout the manuscript: The entire manuscript was carefully rechecked, and statements that were inaccurate, unclear, or inappropriate were revised. These revisions were made throughout the manuscript.

==================================================
**Response to the Academic Editor**
==================================================

**Comment 1**:
Only the PubMed search strategy is reported; full search strings for the other 8 databases should be provided for reproducibility.

Response:
Thank you for this important suggestion. We agree that providing complete database-specific search strategies is essential to ensuring the transparency and reproducibility of the review. We have now provided the complete search strategies for all databases searched in this review, including Web of Science, CINAHL, the Cochrane Library, Embase, China National Knowledge Infrastructure (CNKI), Wanfang Data, the VIP Database, and the China Biomedical Literature Database (CBM). Further details are provided in the supplementary file entitled “Search Strategies.”

--------------------------------------------------

Comment 2:
Two included studies (references 14 and 16) are not gastric-cancer-specific. Authors should clarify inclusion thresholds and flag these findings as lower-weight evidence.

Response:
Thank you for this valuable comment. We have clarified that these two studies were included because they contained gastric cancer patients and provided supplementary evidence regarding symptom network characteristics. However, because gastric cancer patients represented only a subset of the study populations and separate gastric cancer networks were not constructed, these findings were interpreted as indirect evidence with limited applicability.

Location of revision:
Discussion section, Similarities and differences between mixed cancer studies and gastric cancer-specific studies, Page 13, Lines 290–306.

--------------------------------------------------

Comment 3:

Included studies used heterogeneous network analysis methods, which deserves a more critical discussion.

Response:

Thank you for this insightful suggestion. In response to this comment, we carefully reread and reassessed the full texts of all 10 studies initially included, with particular attention to their network construction procedures, network estimation methods, centrality indicators, stability assessments, and reporting characteristics.

During this reassessment, we found that the following study did not provide sufficient details regarding the key components of symptom network analysis:

Wang X, Tong X, Fan H, et al. Application effect of a precision symptom network management program based on enhanced recovery after surgery in older patients with gastric cancer during the perioperative period. Int J Geriatr. 2026;47(2):189–194.

Specifically, this study did not clearly describe the symptom nodes, network edges, network estimation procedures, centrality indicators, bridge symptom analysis, or assessments of network stability and accuracy. Moreover, it did not report the use of R software or another commonly used statistical network analysis platform for network estimation. Therefore, we could not confirm that the study had conducted a formal symptom network analysis consistent with the conceptual eligibility criteria of this scoping review. Consequently, this study was excluded, and the number of included studies was revised from 10 to 9.

In addition, we expanded the Discussion to critically examine the differences among cross-sectional networks, cross-lagged panel networks, longitudinal networks, Gephi-based clustering, and R-based network estimation. We also discussed how variations in network models, centrality indicators, stability assessments, software, and reporting methods may limit the direct comparison of core and bridge symptoms across studies.

Location of revision:

Results section, “Network Analysis Methods and Reporting Characteristics,” Page 10, Lines 208–216.

Discussion section, “Network Analysis Modeling and Heterogeneity in Reporting,” Page 14, Lines 308–327.

The study selection flow diagram, relevant tables, and the number of included studies reported throughout the manuscript were also updated accordingly.

--------------------------------------------------

Comment 4:
There is no explicit statement that risk-of-bias assessment was intentionally omitted.

Response:
Thank you for highlighting this issue. We have explicitly stated that formal risk-of-bias assessment was not conducted because it is not routinely required for scoping reviews. We also acknowledged the limitations caused by heterogeneity in study design, assessment tools, and analytical methods.

Location of revision:
Discussion and Limitations-related statements, Page 15, Lines 347–348.

--------------------------------------------------

Comment 5:
All included studies were from China and South Korea, so cross-cultural generalizability should be discussed.

Response:
Thank you for this suggestion. We have strengthened the discussion regarding geographical limitations and emphasized that symptom network structures require validation in populations with different cultural backgrounds and healthcare systems.

Location of revision:
Conclusion and limitations discussion, Page 15, Lines 339–348.

==================================================
Response to Reviewer #1
==================================================

Comment 1:
Eight of the ten included studies were conducted in China, which may limit generalizability.

Response:
Thank you for this comment. We added a limitation regarding geographical concentration and discussed that symptom network structures, particularly those involving traditional Chinese medicine-related variables, require validation in other populations.

Location:
Conclusion section, Page 15, Lines 339–348.

Comment 2:
Mixed cancer studies may influence synthesis.

Response:
Thank you for this suggestion. We added a dedicated discussion distinguishing mixed cancer studies from gastric cancer-specific studies and clarified that mixed cancer evidence should be interpreted cautiously.

Location:
Discussion section, Page 13, Lines 290–306.

Comment 3:
A descriptive comment on methodological quality would help contextualize evidence strength.

Response:
Thank you. We clarified the rationale for not performing formal risk-of-bias assessment and discussed evidence limitations related to methodological heterogeneity.

Location:
Discussion and Conclusion sections, Page 14–15.

Comment 4:
A schematic figure summarizing central symptoms, bridge symptoms, and pathways was suggested.

Response:
Thank you for this valuable suggestion. Due to substantial heterogeneity in symptom instruments, symptom nodes, network models, and indicators, a pooled network figure may oversimplify findings. Instead, we provided detailed supplementary information and strengthened narrative synthesis.

Location:
Supporting Information section; Results and Discussion sections.

Comment 5:
The manuscript should explain what network analysis adds beyond conventional symptom cluster approaches.

Response:
Thank you. We expanded the Discussion to explain that network analysis provides information on central symptoms, bridge symptoms, and temporal symptom relationships beyond traditional symptom cluster approaches.

Location:
Discussion section, Page 10–12.

==================================================
Response to Reviewer #2
==================================================

Comment 1:
The methodology requires greater detail and transparency.

Response:
Thank you. We clarified the Arksey and O’Malley scoping review framework, screening procedures, and evidence synthesis process. The PRISMA flow diagram was revised to improve transparency.

Location:
Page 3

Comment 2:
The review combines gastric cancer-specific and mixed cancer populations.

Response:
Thank you. We distinguished gastric cancer-specific evidence from mixed cancer evidence and discussed the implications for specificity.

Location:
Discussion, Page 13.

Comment 3:
Fatigue interpretation should be more cautious because network methods differ.

Response:
Thank you. We revised the wording to clarify that fatigue was the most frequently reported core symptom rather than a universally stable symptom across all models.

Location:
Results, Page 7; Discussion, Page 10 and Page 11.

Comment 4:
A supplementary table describing network analysis methods should be added.

Response:
Thank you. We added Table4 summarizing network estimation methods, centrality indicators, bridge symptom analyses, software packages, and reporting characteristics.

Location:
Results section, Page 10.； Discussion, Page 14.

Comment 5:
Clinical intervention conclusions should be moderated.

Response:
Thank you. We revised the Discussion and Conclusions to avoid causal interpretations and emphasize the need for longitudinal and intervention studies.

Location:
Discussion and Conclusion sections, Page 10–15.

==================================================
Response to Reviewer #3
==================================================

Comment 1:
Formal methodological quality or risk-of-bias assessment was suggested.

Response:
Thank you. We explained that formal risk-of-bias assessment was not conducted because it is not mandatory for scoping reviews. We added discussion of evidence limitations.

Location:
Conclusion and limitations-related discussion, Page 15.

Comment 2:
The discussion should analyze heterogeneity among studies.

Response:
Thank you. We expanded discussion of heterogeneity related to study design, symptom assessment tools, measurement time points, and network construction methods.

Location:
Discussion section, Page 10–15.

Comment 3:
The description and critical evaluation of network methods should be expanded.

Response:
Thank you. We added a comprehensive description of network methods and reporting characteristics, including centrality indicators, bridge symptoms, stability assessment, and software.

Location:
Results section, Page 10; Supporting Information S4 Table.

Comment 4:
The discussion of precision intervention should be more cautious.

Response:
Thank you. We moderated the clinical implications and emphasized that identified symptoms represent potential targets rather than confirmed causal intervention points.

Location:
Discussion and Conclusion sections, Page 10–14.

Comment 5:
The generalizability of findings should be further discussed.

Response:
Thank you. We strengthened discussion of geographical limitations and added supplementary materials to improve transparency and reproducibility.

Location:
Conclusion section, Page 15.

==================================================

Response to Journal Requirements

==================================================

We sincerely thank the Editorial Office for outlining these additional requirements. We have carefully checked the revised manuscript and submission files and have addressed each requirement as follows.

1. PLOS ONE style and file-naming requirements

Response:
Thank you for this reminder. We carefully checked the revised manuscript against the PLOS ONE style templates for the main text, title page, author information, and affiliations. We also reviewed and revised the names of the manuscript and Supporting Information files to ensure that they comply with the journal’s formatting and file-naming requirements.

2. Funding information

Response:

Thank you for clarifying the journal’s requirements regarding funding information. We have removed funding-related text from the manuscript, including any such information in the Acknowledgments section or other sections of the main manuscript.

We respectfully request that the Funding Statement in the online submission system be updated to the following:

“JC received funding from the General Program of the Yunnan Provincial Department of Science and Technology (Grant No. 202501AT070122), the Youth Project of the Chinese Nursing Association (Grant No. ZHKYQ202411), and the Yunnan Health Training Project for High-Level Talents (Grant No. H-2025061). The funders had no role in study design, data collection and analysis, decision to publish, or preparation of the manuscript.”

3. Separate captions for all figures

Response:
Thank you for this comment. We have checked all figures and added a separate caption for each figure in the manuscript. The figure captions are clearly listed and correspond to the relevant figure numbers.

4. File type and legends for Supporting Information

Response:

Thank you for pointing this out. We have changed the file type of the supplementary figure from “Figure” to “Supporting Information” in the submission system. We have also ensured that a separate legend for each Supporting Information file is provided in the manuscript after the reference list.
