# Supplementary material for: Application of symptom network analysis among patients with gastric cancer: A scoping review
Source: PLoS One. 2026 Sep 21;21(9):e0358940. doi: 10.1371/journal.pone.0358940 (PMC13592616; doi:10.1371/journal.pone.0358940)
Supplement: Supplementary file 5 [file pone.0358940.s006.docx]

**Response to Reviewers**

**Manuscript title:**

Application of symptom network analysis among patients with gastric cancer: A scoping review

**Manuscript ID:**

PONE-D-26-22137R1

Dear Academic Editor and Reviewers,

We sincerely thank the Academic Editor and Reviewers for the careful reassessment of our manuscript and for the constructive comments provided in this revision round. We have carefully considered each remaining concern and revised the manuscript accordingly. In particular, we added a targeted discussion of the cross-cultural transferability of Traditional Chinese Medicine (TCM) constitution, explicitly contrasted network analysis with conventional symptom cluster approaches in terms of intervention-relevant implications, clarified PRISMA-ScR reporting and the rationale for not conducting a formal methodological quality appraisal, expanded the search strategy, defined key network concepts earlier in the manuscript, strengthened the justification and interpretation of mixed cancer studies, revised causal or overly predictive wording, and expanded the limitations section. Below we provide a point-by-point response and identify the corresponding locations in the revised manuscript.

**==================================================
Response to the Academic Editor
==================================================**

**Comment 1:**

Reviewers still have concerns, and please address.

**Response:**

Thank you for the opportunity to further revise our manuscript. We have addressed all remaining issues raised by Reviewers #1 and #2. Specifically, we added a distinct discussion of the cultural and conceptual transferability of TCM constitution; added a self-contained paragraph explaining how temporal directionality, central symptoms, and bridge symptoms may lead to intervention hypotheses that differ from conventional symptom cluster analysis; clarified compliance with PRISMA-ScR and the intentional omission of formal risk-of-bias/methodological quality appraisal; expanded the search strategy and early definitions of network concepts; clarified the eligibility and limited applicability of mixed cancer studies; revised temporal and TCM-related findings to avoid causal interpretation; and expanded the limitations regarding the small evidence base, geographic concentration, and methodological heterogeneity. We believe these revisions have substantially improved the transparency, conceptual clarity, and methodological caution of the review.

**Location of revision:**

Revisions were made throughout the Abstract, Introduction, Materials and methods, Results, Discussion, and Conclusions, with the main changes located on Pages 2-6 and 10-18 of the revised manuscript.

**==================================================
Response to Reviewer #1
==================================================**

**Comment 1:**

Generalizability of TCM-related findings. I appreciate the expanded discussion of TCM constitution findings in sections 3.3.5 and 4.2, which provide a clearer account of the associations identified between constitution types and specific symptoms. However, my original comment concerned a more specific and, I believe, still unaddressed issue: TCM constitution is a culturally embedded diagnostic construct without direct equivalents in most non-Chinese healthcare systems and assessment frameworks. The current limitations statement addresses cross-cultural generalizability only in general terms ("all were conducted in China or South Korea, which may restrict the cross-cultural generalizability of the findings"), but does not discuss whether, or how, findings involving TCM constitution variables specifically could be expected to translate to, or even be tested in, other cultural and clinical contexts. I would ask the authors to add a brief, targeted discussion on this point, either commenting on the theoretical transferability of the TCM constitution construct itself, or explicitly flagging this as a distinct limitation separate from the review's overall geographic generalizability.

**Response:**

Thank you for clarifying this important distinction. We fully agree that the concern is not only geographic generalizability, but also the conceptual and measurement transferability of the TCM constitution construct itself. We therefore added a dedicated paragraph in Section 4.2 stating that TCM constitution is a culturally embedded diagnostic and classification construct without direct conceptual or measurement equivalents in many non-Chinese healthcare systems. We now explicitly caution that associations involving TCM constitution should not be assumed to generalize to other cultural or clinical settings merely because the symptom nodes themselves are common across populations. We further explain that testing transferability would require explicit cross-cultural operationalization and validation of the constitution construct, followed by replication of the observed symptom-network associations in more diverse populations. In addition, we added this issue as a distinct limitation in the Conclusions, separate from the overall geographic concentration of the included studies. We also further moderated the interpretation of TCM-related findings by describing them as associative and hypothesis-generating rather than causal.

**Location of revision:**

Results, Section 3.3.5 "Association between Traditional Chinese Medicine constitution and symptoms," Pages 10-11; Discussion, Section 4.2 "Limited evidence on bridge symptoms and factors associated with core symptoms," Pages 13-14; Conclusions, Page 18.

--------------------------------------------------

**Comment 2:**

Added value of the network analysis approach over conventional symptom cluster methods. The authors indicated in their response that the Discussion was expanded to address this point; however, I was unable to identify a passage that explicitly contrasts the network analysis approach with conventional symptom cluster methods in terms of the actionable, intervention-relevant differences between the two. The reference to symptom clusters currently remains largely confined to a contextual sentence in the Introduction, without further conceptual development in the Discussion. Given that this distinction represents a central conceptual contribution of the review, I would ask the authors to add a short, self-contained paragraph (a few sentences would suffice) clarifying concretely how (a) the directionality captured in temporal/cross-lagged networks, and (b) the distinction between central and bridge symptoms, translate into intervention strategies that would differ structurally from those derivable from a standard symptom cluster analysis. A concrete example drawn from one of the included studies would be particularly helpful in illustrating this point for readers.

**Response:**

Thank you for this very helpful suggestion. We agree that the previous revision did not make the contrast sufficiently explicit. We have now added a self-contained paragraph in Section 4.3 that directly compares conventional symptom cluster analysis with network analysis. The revised text explains that conventional symptom cluster methods primarily identify groups of co-occurring symptoms and therefore support coordinated management of symptom sets, whereas network analysis can additionally identify high-connectivity central symptoms, bridge symptoms linking symptom communities, and time-ordered associations in longitudinal/cross-lagged models. We then clarify the intervention-relevant implications: central symptoms may serve as candidate high-connectivity targets, bridge symptoms may be considered when the aim is to limit propagation of symptom burden across domains, and time-ordered associations can generate hypotheses about intervention sequencing. To make this distinction concrete, we added an example from Li et al.: altered taste was temporally associated with subsequent loss of appetite. A conventional cluster analysis would support co-management of these eating-related symptoms, whereas the cross-lagged network suggests that early management of taste change could be prospectively tested as an upstream strategy before appetite deterioration. We also note that dry mouth and abdominal distension were identified as bridge symptoms at different chemotherapy stages, illustrating how candidate intervention targets may shift over time. Finally, we explicitly state that these implications remain hypothesis-generating because network centrality, bridge status, and cross-lagged associations do not establish causal intervention effects.

**Location of revision:**

Discussion, Section 4.3 "Limited evidence on longitudinal symptom relationships and potential value of strongly connected symptom pairs for intervention," Page 15 (new self-contained paragraph comparing network analysis with conventional symptom cluster analysis).

**==================================================
Response to Reviewer #2
==================================================**

**Comment 1:**

Please clarify whether the review was conducted and reported in accordance with the PRISMA-ScR guideline and explain whether a methodological quality appraisal was performed or intentionally omitted.

**Response:**

Thank you for highlighting this important reporting issue. We have now explicitly stated in both the Abstract and Methods that the review was reported in accordance with the PRISMA extension for Scoping Reviews (PRISMA-ScR), and we indicate that the completed checklist is provided as Supporting Information (S1 File). We also added a new subsection, Section 2.5 "Reporting guideline and methodological quality appraisal," to explain that a formal risk-of-bias or methodological quality appraisal was intentionally not undertaken because the purpose of this scoping review was to map the breadth, characteristics, methods, and findings of the available evidence rather than to estimate pooled effects or grade certainty of evidence. Instead, methodological features that are directly relevant to interpreting symptom network studies - including network estimation approaches, centrality and bridge indicators, temporal indicators, and stability/accuracy reporting - were systematically charted and summarized in Table 4. We also acknowledge in the limitations that, as a consequence, individual-study methodological quality was not used to weight the synthesis or conclusions.

**Location of revision:**

Abstract, Methods, Page 2; Materials and methods, Section 2.5 "Reporting guideline and methodological quality appraisal," Page 6; Conclusions/limitations, Page 18; Table 4 and S1 File.

--------------------------------------------------

**Comment 2:**

Provide greater detail on the search strategy to improve reproducibility.

**Response:**

Thank you for this suggestion. We expanded Section 2.3 to improve the transparency and reproducibility of the search process. The revised Methods now specify that controlled vocabulary/subject headings and free-text terms were used in combination; synonymous terms within each concept were combined using the Boolean operator OR, while the gastric cancer concept and the symptom network analysis concept were combined using AND. Truncation was applied where appropriate, and the search syntax was adapted according to the indexing and retrieval rules of each database. We also clarified the supplementary snowballing procedure and listed the principal Chinese- and English-language search terms. In addition, the complete PubMed search strategy, including MeSH terms, title/abstract terms, and Boolean combinations, is presented in Table 1. The complete database-specific search strategies for the other eight databases are provided in the supplementary file entitled “Search_Strategies_8_Databases”, which contains the detailed search strings used for each database and can be consulted for further information.

**Location of revision:**

Materials and methods, Section 2.3 "Search strategy," Page 5; Table 1.

--------------------------------------------------

**Comment 3:**

Clearly define key network analysis concepts such as core symptoms and bridge symptoms early in the manuscript.

**Response:**

Thank you for this helpful suggestion. We revised the Introduction to define the basic network terminology before presenting the rationale for the review. We now state that symptoms are represented as nodes and their statistical associations as edges; core symptoms are nodes with relatively high centrality and strong connectivity within the network; and bridge symptoms connect otherwise distinct symptom communities or domains. These definitions are now introduced early so that subsequent Results and Discussion can be interpreted consistently.

**Location of revision:**

Introduction, Page 3 (paragraph introducing symptom network analysis and definitions of nodes, edges, core symptoms, and bridge symptoms).

--------------------------------------------------

**Comment 4:**

The inclusion of mixed cancer populations should be better justified and discussed as a potential limitation.

**Response:**

Thank you. We have strengthened both the eligibility criteria and the interpretation of mixed cancer evidence. In Section 2.2, we now explain that, given the limited evidence base, mixed cancer studies were eligible only when gastric cancer patients were explicitly represented in the sample and the study examined symptom networks in a treatment context relevant to gastric cancer. Because gastric-cancer-specific networks could not be isolated in these studies, their findings were treated as indirect evidence and interpreted separately. The Results now explicitly identify the two mixed cancer studies. In Section 4.4, we compare their findings with gastric-cancer-specific studies and note that gastric cancer patients represented only 62/202 and 83/249 participants, respectively, with no separate gastric cancer subgroup networks. We therefore caution that these findings have limited applicability to gastric cancer populations. This issue is also explicitly listed in the limitations.

**Location of revision:**

Materials and methods, Section 2.2, Pages 4-5; Results, Section 3.2, Page 7; Discussion, Section 4.4, Page 16; Conclusions/limitations, Page 18.

--------------------------------------------------

**Comment 5:**

Interpretations of Traditional Chinese Medicine-related findings and temporal symptom relationships should be presented more cautiously, avoiding causal language where the evidence is observational.

**Response:**

We agree and have systematically revised the manuscript to avoid causal interpretation. In Section 3.3.3, terms such as "direct effect" or overly predictive wording were replaced with "cross-lagged association" or "temporal association," and we explicitly state that these paths should be interpreted as time-ordered statistical associations rather than evidence of causal effects. In Section 3.3.5, TCM constitution findings are described as cross-sectional or time-ordered statistical associations, and we added the statement that these findings are associative and hypothesis-generating and do not demonstrate that TCM constitution causes subsequent symptom changes. The Discussion was revised in the same direction: proposed TCM mechanisms are described as theoretical explanations rather than causal interpretations, and intervention implications based on temporal networks are framed as hypotheses that require prospective evaluation.

**Location of revision:**

Results, Section 3.3.3, Pages 9-10; Results, Section 3.3.5, Pages 10-11; Discussion, Sections 4.2-4.3, Pages 13-15; Conclusions, Pages 17-18.

--------------------------------------------------

**Comment 6:**

Expand the discussion of the review's limitations, including the small number of included studies, predominance of Chinese populations, and methodological heterogeneity across studies.

**Response:**

Thank you for this important suggestion. We substantially expanded the limitations. The revised Conclusions now explicitly state that only nine studies were included; seven were conducted in China and two in South Korea, limiting geographic and cross-cultural generalizability; two studies involved mixed cancer populations and did not provide gastric-cancer-specific networks; and TCM constitution has additional conceptual and measurement transferability limitations outside Chinese contexts. We also describe the considerable methodological heterogeneity in study design, symptom assessment tools, assessment time points, node selection, network estimation methods, centrality measures, and reporting of stability and accuracy. In addition, Section 4.5 now critically discusses how different network models, algorithms, centrality metrics, regularization choices, and incomplete stability/accuracy reporting may affect the comparability of core and bridge symptom findings. Finally, we explicitly acknowledge that the lack of formal methodological quality appraisal means that individual-study quality was not used to weight the synthesis or conclusions.

**Location of revision:**

Discussion, Section 4.5 "Network analysis modeling and heterogeneity in reporting," Pages 16-17; Conclusions/limitations, Page 18.

**==================================================
Response to Reviewer #3
==================================================**

**Comment 1:**

I thank the authors for their careful revision. The comments from the previous review have been adequately addressed, and the manuscript has improved accordingly. I have no further comments and consider it suitable for publication.

**Response:**

We sincerely thank Reviewer #3 for the positive assessment of our revision and for the constructive comments provided during the previous review round. We greatly appreciate the Reviewer's recognition that the manuscript has improved and that the previous concerns have been adequately addressed. No additional revision was required specifically in response to this comment; however, we have further refined the manuscript in response to the remaining comments from the Academic Editor and the other reviewers.

==================================================
Response to Journal Requirements
==================================================

Requirement 1:

Any changes to the reference list should be mentioned in the rebuttal letter that accompanies the revised manuscript.

Response:

Thank you for this reminder. We carefully rechecked the reference list against all in-text citations and made the following changes. First, we removed Huang Y, Wang H, Li Y, Xiao X. “Symptom clusters in gastric cancer patients receiving chemotherapy: a scoping review.” Chin Gen Pract. 2025;28(26):3338–44 (former Reference 18), because this entry was included in the previous reference list but was not cited in the manuscript; removing it ensures consistency between the text and the reference list. Second, to support the newly added paragraph that explicitly contrasts conventional symptom cluster analysis with network analysis, we added Miaskowski C, Barsevick A, Berger A, Casagrande R, Grady PA, Jacobsen P, et al. “Advancing Symptom Science Through Symptom Cluster Research: Expert Panel Proceedings and Recommendations.” J Natl Cancer Inst. 2017;109(4):djw253, as current Reference 23. Third, two existing references were moved earlier in the reference list because they are now cited in the Introduction to support newly added definitions: Robinaugh et al. was moved from former Reference 17 to current Reference 5 to support the definition of core symptoms, and Luo et al. was moved from former Reference 19 to current Reference 6 to support the definition of bridge symptoms. The numbering of all subsequent in-text citations and reference-list entries was updated accordingly. We also rechecked the entire manuscript to ensure consistency between all in-text citations and the final reference list.

Location of revision:

Introduction, Page 3; Discussion, Section 4.3, Page 15; References, Pages 19-23.

**Closing statement**

We again thank the Academic Editor and all Reviewers for their careful and constructive evaluation. We believe that the revisions made in response to these comments have improved the methodological transparency, interpretive caution, and clinical relevance of the manuscript. We hope that the revised manuscript is now suitable for publication in PLOS ONE.
