# Supplementary material for: Application of symptom network analysis among patients with gastric cancer: A scoping review
Source: PLoS One. 2026 Sep 21;21(9):e0358940. doi: 10.1371/journal.pone.0358940 (PMC13592616; doi:10.1371/journal.pone.0358940)
Supplement: Supplementary file 6 [file pone.0358940.s001.docx]

Fig 1.Flowchart of literature screening.
