# Supplementary material for: Application of symptom network analysis among patients with gastric cancer: A scoping review
Source: PLoS One. 2026 Sep 21;21(9):e0358940. doi: 10.1371/journal.pone.0358940 (PMC13592616; doi:10.1371/journal.pone.0358940)
Supplement: Peer review history

# Peer review: Decision Letter 0

**DOI:** 10.1371/journal.pone.0358940.r001

**Authors:** Jincheng Wang

14 Jul 2026

Dear Dr. Zhu,

Thank you for submitting your manuscript to PLOS ONE. After careful consideration, we feel that it has merit but does not fully meet PLOS ONE’s publication criteria as it currently stands. Therefore, we invite you to submit a revised version of the manuscript that addresses the points raised during the review process.

==============================

**ACADEMIC EDITOR:** Only the PubMed search strategy is reported; full search strings for the other 8 databases should be provided for reproducibility. Two included studies (refs 14, 16) are not gastric-cancer-specific (only 62/202 and 83/249 patients had gastric cancer), so authors should clarify inclusion thresholds and flag these findings as lower-weight in the synthesis. Included studies used heterogeneous network analysis methods (cross-sectional, cross-lagged panel, Gephi-based clustering vs. R-based networks), which is only briefly acknowledged and deserves a more critical discussion of how it limits cross-study comparability of "central/bridge symptoms." There is no explicit statement that risk-of-bias assessment was intentionally omitted. Finally, all 10 studies are from China/South Korea, so the limitation on cross-cultural generalizability should be stated more explicitly.

==============================

Please submit your revised manuscript by Aug 28 2026 11:59PM. If you will need more time than this to complete your revisions, please reply to this message or contact the journal office at plosone@plos.org. When you're ready to submit your revision, log on to https://www.editorialmanager.com/pone/ and select the 'Submissions Needing Revision' folder to locate your manuscript file.

- A letter that responds to each point raised by the academic editor and reviewer(s). You should upload this letter as a separate file labeled 'Response to Reviewers'.
- A marked-up copy of your manuscript that highlights changes made to the original version. You should upload this as a separate file labeled 'Revised Manuscript with Track Changes'.
- An unmarked version of your revised paper without tracked changes. You should upload this as a separate file labeled 'Manuscript'.

If you would like to make changes to your financial disclosure, please include your updated statement in your cover letter. Guidelines for resubmitting your figure files are available below the reviewer comments at the end of this letter.

If applicable, we recommend that you deposit your laboratory protocols in protocols.io to enhance the reproducibility of your results. Protocols.io assigns your protocol its own identifier (DOI) so that it can be cited independently in the future. For instructions see: https://journals.plos.org/plosone/s/submission-guidelines#loc-laboratory-protocols. Additionally, PLOS ONE offers an option for publishing peer-reviewed Lab Protocol articles, which describe protocols hosted on protocols.io. Read more information on sharing protocols at https://plos.org/protocols?utm_medium=editorial-email&utm_source=authorletters&utm_campaign=protocols.

As the corresponding author, your ORCID iD is verified in the submission system and will appear in the published article. PLOS supports the use of ORCID, and we encourage all coauthors to register for an ORCID iD and use it as well. Please encourage your coauthors to verify their ORCID iD within the submission system before final acceptance, as unverified ORCID iDs will not appear in the published article. *Only*  the individual author can complete the verification step; PLOS staff *cannot*  verify ORCID iDs on behalf of authors.

We look forward to receiving your revised manuscript.

Kind regards,

Jincheng Wang

Academic Editor

PLOS One

Journal requirements:

When submitting your revision, we need you to address these additional requirements.

1. Please ensure that your manuscript meets PLOS ONE's style requirements, including those for file naming. The PLOS ONE style templates can be found at

https://journals.plos.org/plosone/s/file?id=wjVg/PLOSOne_formatting_sample_main_body.pdf and

https://journals.plos.org/plosone/s/file?id=ba62/PLOSOne_formatting_sample_title_authors_affiliations.pdf

2. Thank you for stating the following in the Funding Section of your manuscript:

“This work was supported by the Youth Project of the Chinese Nursing Association (No. ZHKYQ202411), the General Program of the Yunnan Provincial Department of Science and Technology (No. 202501AT070122), and the Scientific Research Fund of the Yunnan Provincial Department of Education (No. 2026Y0619).”

We note that you have provided funding information that is currently declared in your Funding Statement. However, funding information should not appear in the Acknowledgments section or other areas of your manuscript. We will only publish funding information present in the Funding Statement section of the online submission form.

Please remove any funding-related text from the manuscript and let us know how you would like to update your Funding Statement. Currently, your Funding Statement reads as follows:

“JC received funding from the Youth Project of the Chinese Nursing Association (Grant No. ZHKYQ202411; https://www.zhhlxh.org.cn/), the General Program of the Yunnan Provincial Department of Science and Technology (Grant No. 202501AT070122; https://kjt.yn.gov.cn/), and the Scientific Research Fund of the Yunnan Provincial Department of Education (Grant No. 2026Y0619; https://jyt.yn.gov.cn/). The funders had no role in study design, data collection and analysis, decision to publish, or preparation of the manuscript.”

Please include your amended statements within your cover letter; we will change the online submission form on your behalf.

3. PLOS requires an ORCID iD for the corresponding author in Editorial Manager on papers submitted after December 6th, 2016. Please ensure that you have an ORCID iD and that it is validated in Editorial Manager. To do this, go to ‘Update my Information’ (in the upper left-hand corner of the main menu), and click on the Fetch/Validate link next to the ORCID field. This will take you to the ORCID site and allow you to create a new iD or authenticate a pre-existing iD in Editorial Manager.

4. Please include a separate caption for each figure in your manuscript.

5. We notice that your supplementary figure is uploaded with the file type 'Figure'. Please amend the file type to 'Supporting Information'. Please ensure that each Supporting Information file has a legend listed in the manuscript after the references list.

If the reviewer comments include a recommendation to cite specific previously published works, please review and evaluate these publications to determine whether they are relevant and should be cited. There is no requirement to cite these works unless the editor has indicated otherwise.

Additional Editor Comments:

Only the PubMed search strategy is reported; full search strings for the other 8 databases should be provided for reproducibility. Two included studies (refs 14, 16) are not gastric-cancer-specific (only 62/202 and 83/249 patients had gastric cancer), so authors should clarify inclusion thresholds and flag these findings as lower-weight in the synthesis. Included studies used heterogeneous network analysis methods (cross-sectional, cross-lagged panel, Gephi-based clustering vs. R-based networks), which is only briefly acknowledged and deserves a more critical discussion of how it limits cross-study comparability of "central/bridge symptoms." There is no explicit statement that risk-of-bias assessment was intentionally omitted. Finally, all 10 studies are from China/South Korea, so the limitation on cross-cultural generalizability should be stated more explicitly.

[Note: HTML markup is below. Please do not edit.]

Reviewers' comments:

Reviewer's Responses to Questions

**Comments to the Author**

1. Is the manuscript technically sound, and do the data support the conclusions?

Reviewer #1: Yes

Reviewer #2: Yes

Reviewer #3: Partly

**********

2. Has the statistical analysis been performed appropriately and rigorously?

Reviewer #1: Yes

Reviewer #2: Yes

Reviewer #3: Yes

**********

3. Have the authors made all data underlying the findings in their manuscript fully available?

The PLOS Data policy

Reviewer #1: Yes

Reviewer #2: Yes

Reviewer #3: Yes

**********

4. Is the manuscript presented in an intelligible fashion and written in standard English?

Reviewer #1: Yes

Reviewer #2: Yes

Reviewer #3: Yes

**********

Reviewer #1: This scoping review systematically maps the available evidence on symptom network analysis in patients with gastric cancer, with the aim of identifying central and bridge symptoms, key symptom pathways, temporal relationships, and influencing factors. Following a search of nine databases up to April 2026, ten studies were included: five cross-sectional, four longitudinal, and one quasi-experimental, predominantly conducted in China. The most frequently used assessment instruments were the MDASI and the MDASI-GI-C. The review identifies fatigue as the most consistent central symptom across treatment phases, highlights the gastrointestinal symptom chain (altered taste perception, appetite loss, nausea, and vomiting) as the most disease-specific network pathway, and underscores the bridging role of psychological symptoms such as distress, sadness, and anxiety. The authors conclude that symptom management in gastric cancer should move beyond single-symptom control toward precision interventions targeting key network nodes, and call for future longitudinal studies with stratified designs and intervention-focused outcomes.

Minor Comments

1 Eight of the ten included studies were conducted in China, which naturally reflects the current state of the literature but may limit the generalizability of the findings to other populations and healthcare contexts. It would strengthen the manuscript if the authors briefly acknowledged this in the limitations section and discussed whether the identified symptom network structures, particularly those involving TCM-related variables, are expected to be reproducible in non-Chinese cohorts.

2 Two included studies (Wang et al. [14] and Rha et al. [16]) were not conducted exclusively in gastric cancer patients, with gastric cancer subsamples of 62 and 83 patients respectively. The authors acknowledge this in the conclusions, which is appreciated. It may be worth adding a brief comment in the limitations section on how the inclusion of these mixed-population studies could have influenced the synthesis, particularly given that symptom network structures may differ across cancer types.

3 The authors correctly note that scoping reviews do not require formal risk of bias assessment. However, given the small number of included studies and the considerable heterogeneity in design, assessment tools, and analytical methods, a brief descriptive comment on the overall methodological quality of the evidence, even informal, would help readers contextualize the strength of the conclusions. This could be addressed concisely within the limitations section.

4 Given that the central contribution of this review concerns network structure and topology, the inclusion of at least one schematic figure summarizing the most consistently identified central symptoms, bridge symptoms, and key pathways across studies would considerably improve clarity and accessibility for readers. This is of course at the authors' discretion, but it would make the clinical implications more immediately interpretable.

5 The clinical recommendations are well-grounded and appropriate. As a minor suggestion, the authors might consider briefly elaborating on what the network analysis framework specifically adds over conventional symptom cluster approaches, for instance, how actionable directionality in temporal networks or the distinction between central and bridge symptoms translates into structurally different intervention strategies. Even a few sentences on this point would reinforce the conceptual contribution of the review.

Reviewer #2: 1. The methodology requires greater detail and transparency. Although the review follows a scoping review approach, the authors should explicitly state the methodological framework used (e.g., JBI or Arksey and O'Malley framework) and provide a more detailed description of the evidence synthesis process. In addition, the PRISMA flow diagram should clearly report the number of duplicates removed, full-text articles assessed, and reasons for exclusion.

2. The review combines evidence from studies exclusively involving gastric cancer patients with studies including mixed digestive cancer or mixed cancer populations. This may affect the specificity of the conclusions.

3.The authors should clearly distinguish findings derived from gastric cancer cohorts from those obtained from broader cancer populations and discuss the potential impact on generalizability.

The discussion emphasizes fatigue as the most stable central symptom across treatment phases. While this observation is supported by several included studies, the interpretation should be more cautious because network methodologies, symptom assessment tools, and centrality metrics varied considerably across studies. The authors should acknowledge that direct comparisons of central symptoms across different network models may be challenging.

4. The review would benefit from a more comprehensive summary of the network analysis methods used in the included studies. A supplementary table describing the network estimation approach, centrality indices, bridge symptom analyses, and software packages used would substantially enhance the scientific value of the review and help readers interpret differences across studies.

5. The conclusions regarding clinical intervention targets should be moderated. Most included studies were cross-sectional, limiting causal inference. While symptoms such as fatigue, altered taste perception, appetite loss, and distress appear to be promising intervention targets, future longitudinal and interventional studies are needed before definitive recommendations can be made.

Reviewer #3: I would like to thank the authors for the opportunity to review this interesting manuscript. I greatly enjoyed reading the paper and appreciate the considerable effort invested in this work. In my view, this review provides a valuable perspective on the complexity of symptom interactions within this population and has the potential to advance both research and clinical practice in the field.

With the sole intention of further strengthening the manuscript, I would like to offer the following comments and suggestions:

First, I believe it would be beneficial to include a formal assessment of methodological quality or risk of bias for the studies included in the review. Such an evaluation would provide important context regarding the strength and reliability of the evidence supporting the findings and their clinical implications.

Second, the discussion could be enhanced by a more in-depth examination of the heterogeneity across the included studies, particularly with respect to study design, assessment instruments, timing of measurements, and the analytical approaches used to construct symptom networks.

Given that network analysis represents the central focus of this review, I also encourage the authors to expand their description and critical appraisal of the methodologies employed in the included studies. This could include a more detailed discussion of centrality metrics, bridge symptoms, network stability, and validation procedures, all of which are essential for interpreting network-based findings.

In addition, I recommend adopting a more cautious tone when discussing the implementation of precision interventions. Since the majority of the evidence synthesized in this review is derived from observational studies, a more balanced interpretation would better align the conclusions with the current level of evidence.

Finally, it would be valuable to further discuss the generalizability of the findings to populations beyond those represented in the included studies. The addition of supplementary materials that enhance transparency and reproducibility would also strengthen the overall contribution of the review.

I would like to thank the authors once again for their contribution to this important area of research. I hope these comments prove helpful in refining an already valuable manuscript with meaningful potential impact for both the scientific and clinical communities.

**********

what does this mean?). If published, this will include your full peer review and any attached files.

If you choose “no”, your identity will remain anonymous but your review may still be made public.

**Do you want your identity to be public for this peer review?**  For information about this choice, including consent withdrawal, please see our Privacy Policy

Reviewer #1: No

Reviewer #2: **Yes:**  Sakarie Mustafe Hidig,MD

Reviewer #3: No

**********

[NOTE: If reviewer comments were submitted as an attachment file, they will be attached to this email and accessible via the submission site. Please log into your account, locate the manuscript record, and check for the action link "View Attachments". If this link does not appear, there are no attachment files.]

To ensure your figures meet our technical requirements, please review our figure guidelines: https://journals.plos.org/plosone/s/figures

You may also use PLOS’s free figure tool, NAAS, to help you prepare publication quality figures: https://journals.plos.org/plosone/s/figures#loc-tools-for-figure-preparation.

NAAS will assess whether your figures meet our technical requirements by comparing each figure against our figure specifications.

# Author comment: Author response to Decision Letter 1

**DOI:** 10.1371/journal.pone.0358940.r002

22 Jul 2026

Please see the uploaded “Response to Reviewers” document for our detailed point-by-point responses to the Academic Editor and all reviewers. All revisions have been incorporated into both the clean manuscript and the marked-up revised manuscript.

Attachment Submitted filename: Response to Reviewers.docx

# Peer review: Decision Letter 1

**DOI:** 10.1371/journal.pone.0358940.r003

**Authors:** Jincheng Wang

6 Aug 2026

Dear Dr. Zhu,

Thank you for submitting your manuscript to PLOS ONE. After careful consideration, we feel that it has merit but does not fully meet PLOS ONE’s publication criteria as it currently stands. Therefore, we invite you to submit a revised version of the manuscript that addresses the points raised during the review process.

==============================

**ACADEMIC EDITOR:**  Reviewers still have concerns, and please address.

==============================

Please submit your revised manuscript by Sep 05 2026 11:59PM. If you will need more time than this to complete your revisions, please reply to this message or contact the journal office at plosone@plos.org. When you're ready to submit your revision, log on to https://www.editorialmanager.com/pone/ and select the 'Submissions Needing Revision' folder to locate your manuscript file.

- A letter that responds to each point raised by the academic editor and reviewer(s). You should upload this letter as a separate file labeled 'Response to Reviewers'.
- A marked-up copy of your manuscript that highlights changes made to the original version. You should upload this as a separate file labeled 'Revised Manuscript with Track Changes'.
- An unmarked version of your revised paper without tracked changes. You should upload this as a separate file labeled 'Manuscript'.

If you would like to make changes to your financial disclosure, please include your updated statement in your cover letter. Guidelines for resubmitting your figure files are available below the reviewer comments at the end of this letter.

If applicable, we recommend that you deposit your laboratory protocols in protocols.io to enhance the reproducibility of your results. Protocols.io assigns your protocol its own identifier (DOI) so that it can be cited independently in the future. For instructions see: https://journals.plos.org/plosone/s/submission-guidelines#loc-laboratory-protocols. Additionally, PLOS ONE offers an option for publishing peer-reviewed Lab Protocol articles, which describe protocols hosted on protocols.io. Read more information on sharing protocols at https://plos.org/protocols?utm_medium=editorial-email&utm_source=authorletters&utm_campaign=protocols.

As the corresponding author, your ORCID iD is verified in the submission system and will appear in the published article. PLOS supports the use of ORCID, and we encourage all coauthors to register for an ORCID iD and use it as well. Please encourage your coauthors to verify their ORCID iD within the submission system before final acceptance, as unverified ORCID iDs will not appear in the published article. *Only*  the individual author can complete the verification step; PLOS staff *cannot*  verify ORCID iDs on behalf of authors.

We look forward to receiving your revised manuscript.

Kind regards,

Jincheng Wang

Academic Editor

PLOS One

Journal Requirements:

If the reviewer comments include a recommendation to cite specific previously published works, please review and evaluate these publications to determine whether they are relevant and should be cited. There is no requirement to cite these works unless the editor has indicated otherwise.

Please review your reference list to ensure that it is complete and correct. If you have cited papers that have been retracted, please include the rationale for doing so in the manuscript text, or remove these references and replace them with relevant current references. Any changes to the reference list should be mentioned in the rebuttal letter that accompanies your revised manuscript. If you need to cite a retracted article, indicate the article’s retracted status in the References list and also include a citation and full reference for the retraction notice.

Additional Editor Comments:

Reviewers still have concerns, and please address.

[Note: HTML markup is below. Please do not edit.]

Reviewers' comments:

Reviewer's Responses to Questions

**Comments to the Author**

Reviewer #1: (No Response)

Reviewer #2: (No Response)

Reviewer #3: All comments have been addressed

**********

2. Is the manuscript technically sound, and do the data support the conclusions?

Reviewer #1: Partly

Reviewer #2: Yes

Reviewer #3: Yes

**********

3. Has the statistical analysis been performed appropriately and rigorously?

Reviewer #1: No

Reviewer #2: Yes

Reviewer #3: Yes

**********

4. Have the authors made all data underlying the findings in their manuscript fully available?

The PLOS Data policy

Reviewer #1: Yes

Reviewer #2: Yes

Reviewer #3: Yes

**********

5. Is the manuscript presented in an intelligible fashion and written in standard English?

Reviewer #1: Yes

Reviewer #2: Yes

Reviewer #3: Yes

**********

Reviewer #1: I thank the authors for their thorough response to the first round of comments. Most points have been adequately addressed. However, I would like to follow up on two comments that I do not believe have been fully resolved in the revised manuscript.

1. Generalizability of TCM-related findings

I appreciate the expanded discussion of TCM constitution findings in sections 3.3.5 and 4.2, which provide a clearer account of the associations identified between constitution types and specific symptoms. However, my original comment concerned a more specific and, I believe, still unaddressed issue: TCM constitution is a culturally embedded diagnostic construct without direct equivalents in most non-Chinese healthcare systems and assessment frameworks. The current limitations statement addresses cross-cultural generalizability only in general terms ("all were conducted in China or South Korea, which may restrict the cross-cultural generalizability of the findings"), but does not discuss whether, or how, findings involving TCM constitution variables specifically could be expected to translate to, or even be tested in, other cultural and clinical contexts. I would ask the authors to add a brief, targeted discussion on this point, either commenting on the theoretical transferability of the TCM constitution construct itself, or explicitly flagging this as a distinct limitation separate from the review's overall geographic generalizability.

2. Added value of the network analysis approach over conventional symptom cluster methods

The authors indicated in their response that the Discussion was expanded to address this point; however, I was unable to identify a passage that explicitly contrasts the network analysis approach with conventional symptom cluster methods in terms of the actionable, intervention-relevant differences between the two. The reference to symptom clusters currently remains largely confined to a contextual sentence in the Introduction, without further conceptual development in the Discussion. Given that this distinction represents a central conceptual contribution of the review, I would ask the authors to add a short, self-contained paragraph (a few sentences would suffice) clarifying concretely how (a) the directionality captured in temporal/cross-lagged networks, and (b) the distinction between central and bridge symptoms, translate into intervention strategies that would differ structurally from those derivable from a standard symptom cluster analysis. A concrete example drawn from one of the included studies would be particularly helpful in illustrating this point for readers.

Reviewer #2: The authors present a timely and clinically relevant scoping review on symptom network analysis in patients with gastric cancer. The manuscript is generally well organized and provides a useful synthesis of the current evidence. However, several issues should be addressed to strengthen the review. First, please clarify whether the review was conducted and reported in accordance with the PRISMA-ScR guideline and explain whether a methodological quality appraisal was performed or intentionally omitted. Second, provide greater detail on the search strategy to improve reproducibility. Third, clearly define key network analysis concepts such as core symptoms and bridge symptoms early in the manuscript. The inclusion of mixed cancer populations should be better justified and discussed as a potential limitation. In addition, interpretations of Traditional Chinese Medicine-related findings and temporal symptom relationships should be presented more cautiously, avoiding causal language where the evidence is observational. Finally, expand the discussion of the review’s limitations, including the small number of included studies, predominance of Chinese populations, and methodological heterogeneity across studies. Addressing these points will improve the clarity, transparency, and clinical value of the manuscript.

Reviewer #3: I thank the authors for their careful revision. The comments from the previous review have been adequately addressed, and the manuscript has improved accordingly. I have no further comments and consider it suitable for publication.

**********

what does this mean?). If published, this will include your full peer review and any attached files.

If you choose “no”, your identity will remain anonymous but your review may still be made public.

**Do you want your identity to be public for this peer review?**  For information about this choice, including consent withdrawal, please see our Privacy Policy

Reviewer #1: No

Reviewer #2: **Yes:**  Dr. Sakarie Mustafe Hidig, MD

Reviewer #3: No

**********

[NOTE: If reviewer comments were submitted as an attachment file, they will be attached to this email and accessible via the submission site. Please log into your account, locate the manuscript record, and check for the action link "View Attachments". If this link does not appear, there are no attachment files.]

To ensure your figures meet our technical requirements, please review our figure guidelines: https://journals.plos.org/plosone/s/figures

You may also use PLOS’s free figure tool, NAAS, to help you prepare publication quality figures: https://journals.plos.org/plosone/s/figures#loc-tools-for-figure-preparation.

NAAS will assess whether your figures meet our technical requirements by comparing each figure against our figure specifications.

# Author comment: Author response to Decision Letter 2

**DOI:** 10.1371/journal.pone.0358940.r004

11 Aug 2026

Dear Academic Editor and Reviewers,

Thank you very much for your careful reassessment of our manuscript and for the constructive comments provided in this revision round. We have carefully addressed all remaining concerns and revised the manuscript accordingly.

In response to the Academic Editor’s request, we have addressed all outstanding issues raised by Reviewers #1 and #2.

For Reviewer #1, we added a dedicated discussion of the cross-cultural transferability of Traditional Chinese Medicine (TCM) constitution, emphasizing that it is a culturally embedded diagnostic and classification construct without direct conceptual or measurement equivalents in many non-Chinese healthcare systems. We also explicitly identified this issue as a distinct limitation. In addition, we added a self-contained paragraph comparing symptom network analysis with conventional symptom cluster analysis, explaining the intervention-relevant implications of central symptoms, bridge symptoms, and time-ordered associations in longitudinal/cross-lagged networks, together with a concrete example from an included study.

For Reviewer #2, we clarified that the review was reported in accordance with PRISMA-ScR and explained that formal methodological quality/risk-of-bias appraisal was intentionally not undertaken because the purpose of this scoping review was to map the available evidence. We expanded the search strategy to improve reproducibility and provided complete database-specific search strategies for the other eight databases in the supplementary file “Search_Strategies_8_Databases.” We also defined core and bridge symptoms earlier in the Introduction, strengthened the eligibility criteria and cautious interpretation of mixed cancer studies, revised TCM-related and temporal findings to avoid causal language, and substantially expanded the limitations regarding the small number of included studies, geographic concentration, mixed cancer populations, cross-cultural transferability, and methodological heterogeneity.

We sincerely thank Reviewer #3 for the positive assessment and for considering the revised manuscript suitable for publication.

All corresponding changes have been incorporated into the revised manuscript. A complete point-by-point response, including the specific locations of each revision, has also been uploaded separately as the “Response to Reviewers” file.

Thank you again for your careful consideration.

Attachment Submitted filename: Response_to_Reviewers.docx

# Peer review: Decision Letter 2

**DOI:** 10.1371/journal.pone.0358940.r005

**Authors:** Jincheng Wang

8 Sep 2026

Application of symptom network analysis among patients with gastric cancer: A scoping review

PONE-D-26-22137R2

Dear Dr. Zhu,

We’re pleased to inform you that your manuscript has been judged scientifically suitable for publication and will be formally accepted for publication once it meets all outstanding technical requirements.

Within one week, you’ll receive an e-mail detailing the required amendments. When these have been addressed, you’ll receive a formal acceptance letter and your manuscript will be scheduled for publication.

An invoice will be generated when your article is formally accepted. Please note, if your institution has a publishing partnership with PLOS and your article meets the relevant criteria, all or part of your publication costs will be covered. Please make sure your user information is up-to-date by logging into Editorial Manager at Editorial Manager® and clicking the ‘Update My Information' link at the top of the page. For questions related to billing, please contact billing support.

If your institution or institutions have a press office, please notify them about your upcoming paper to help maximize its impact. If they’ll be preparing press materials, please inform our press team as soon as possible -- no later than 48 hours after receiving the formal acceptance. Your manuscript will remain under strict press embargo until 2 pm Eastern Time on the date of publication. For more information, please contact onepress@plos.org.

Kind regards,

Jincheng Wang

Academic Editor

PLOS One

Additional Editor Comments (optional):

Reviewers' comments:

Reviewer's Responses to Questions

**Comments to the Author**

Reviewer #2: All comments have been addressed

Reviewer #3: All comments have been addressed

**********

2. Is the manuscript technically sound, and do the data support the conclusions?

Reviewer #2: Yes

Reviewer #3: Yes

**********

3. Has the statistical analysis been performed appropriately and rigorously?

Reviewer #2: Yes

Reviewer #3: Yes

**********

4. Have the authors made all data underlying the findings in their manuscript fully available?

The PLOS Data policy

Reviewer #2: Yes

Reviewer #3: Yes

**********

5. Is the manuscript presented in an intelligible fashion and written in standard English?

Reviewer #2: Yes

Reviewer #3: Yes

**********

Reviewer #2: The authors have addressed all of my comments and concerns raised during the previous review. The manuscript has been substantially improved, with the revisions enhancing its overall clarity, structure, and scientific quality. The responses provided by the authors are satisfactory, and the requested changes have been appropriately incorporated into the revised manuscript. I appreciate the authors’ efforts in carefully considering the reviewers’ suggestions and making the necessary amendments. In its current form, the manuscript has addressed the major issues identified in the previous round of review and is significantly improved. I have no further major comments or concerns regarding the revised manuscript.

Reviewer #3: I thank the authors for carefully addressing the previous comments. The revised manuscript is clear, methodologically appropriate, and presents conclusions consistent with the available evidence. The study limitations and associative nature of the findings are appropriately acknowledged. I consider the manuscript suitable for publication in PLOS ONE and have no further comments.

**********

what does this mean?). If published, this will include your full peer review and any attached files.

If you choose “no”, your identity will remain anonymous but your review may still be made public.

**Do you want your identity to be public for this peer review?**  For information about this choice, including consent withdrawal, please see our Privacy Policy

Reviewer #2: **Yes:**  Sakarie Mustafe Hidig,MD

Reviewer #3: **Yes:**  SERGIO AYALA-DIAZ

**********

# Editor report: Acceptance letter

**DOI:** 10.1371/journal.pone.0358940.r006

**Authors:** Jincheng Wang

PONE-D-26-22137R2

PLOS One

Dear Dr. Zhu,

I'm pleased to inform you that your manuscript has been deemed suitable for publication in PLOS One. Congratulations! Your manuscript is now being handed over to our production team.

At this stage, our production department will prepare your paper for publication. This includes ensuring the following:

* All references, tables, and figures are properly cited

* All relevant supporting information is included in the manuscript submission,

* There are no issues that prevent the paper from being properly typeset

You will receive further instructions from the production team, including instructions on how to review your proof when it is ready. Please keep in mind that we are working through a large volume of accepted articles, so please give us a few days to review your paper and let you know the next and final steps.

Lastly, if your institution or institutions have a press office, please let them know about your upcoming paper now to help maximize its impact. If they'll be preparing press materials, please inform our press team within the next 48 hours. Your manuscript will remain under strict press embargo until 2 pm Eastern Time on the date of publication. For more information, please contact onepress@plos.org.

You will receive an invoice from PLOS for your publication fee after your manuscript has reached the completed accept phase. If you receive an email requesting payment before acceptance or for any other service, this may be a phishing scheme. Learn how to identify phishing emails and protect your accounts at https://explore.plos.org/phishing.

If we can help with anything else, please email us at customercare@plos.org.

Thank you for submitting your work to PLOS One and supporting open access.

Kind regards,

PLOS One Editorial Office Staff

on behalf of

Dr. Jincheng Wang

Academic Editor

PLOS One
